# Localization of damage in the human leg muscles induced by downhill running

**DOI:** 10.1038/s41598-017-06129-8

**Published:** 2017-07-18

**Authors:** Sumiaki Maeo, Yukino Ando, Hiroaki Kanehisa, Yasuo Kawakami

**Affiliations:** 1 Faculty of Sport Sciences, Waseda University, Tokorozawa, Saitama Japan; 2 Research Fellow of Japan Society for the Promotion of Science, Chiyoda, Tokyo Japan; 3 School of Sport, Exercise and Health Sciences, Loughborough University, Loughborough, Leicestershire United Kingdom; 4 Department of Sports and Life Science, National Institute of Fitness and Sports in Kanoya, Kanoya, Kagoshima Japan

## Abstract

We investigated localization of damage within the knee extensors (KEs) and plantar flexors (PFs) induced by downhill running (DR) by using transverse relaxation time (T_2_)-weighted magnetic resonance imaging (MRI). Fourteen young adults performed 45-min DR (−15% slope) at their maximal tolerable velocity. At pre- and 24, 48, and 72 h post-exercise, T_2_-MRI was scanned and T_2_ values for each muscle composing KEs and PFs at proximal, middle, and distal sites were calculated. Maximal isometric torque and rate of torque development (RTD: 0–30, 0–50, 0–100, 0–200 ms) were also measured. Maximal torque significantly decreased in KEs (14–17%) and PFs (6–8%) at 24–48 h post-exercise, with greater reductions for KEs. RTD in all phases, except for 0–200 ms in PFs, significantly decreased in KEs (11–42%) and PFs (13–23%) at least at one time point post-exercise. T_2_ significantly increased at several sites (3–5%) in both muscle groups at 24 and/or 48 h post-exercise. Among the T_2_-increased sites, the peak effect size (Cohen’s *d*) regarding T_2_ change was pronounced at proximal (1.05) and middle (1.64) vastus intermedius compared to the other sites (0.72–0.77). These results suggest that DR induces damage in both KEs and PFs, and especially affects proximal–middle sites of the vastus intermedius.

## Introduction

Trail running and some marathon races involve not only level but also uphill and downhill running (DR)^1^. These sports have become very popular, and trail running especially has seen an exponential increase in worldwide popularity over recent years^1, 2^. Compared to level running, uphill running and DR are typically characterized as a concentrically (muscle shortening) and eccentrically (muscle lengthening) biased exercise, respectively^1, 2^. It is often reported that trail running and mountain ultra-marathon races induce muscle damage in leg muscles, demonstrating as strength loss, occurrence of delayed onset muscle soreness (DOMS), and increased plasma creatine kinase (CK) activity, which could take several days to recover^2–5^. While exercise-induced strength loss can originate at central and/or peripheral levels^6^, central changes recover quickly from a fatigued state^4, 7, 8^ (as early as within few seconds^7^), and do not last for a few days even after performing >30 h of mountain ultra-marathon^4^. Thus, it is suggested that prolonged strength loss lasting for several days, as well as increased DOMS and CK that usually peak 1–3 days post-exercise, can be mainly attributed to muscle damage (i.e. peripheral change)^9–12^. It is known that muscle damage is typically induced by eccentrically biased exercise such as DR. A plausible mechanism for such damage is often attributed to high active muscle lengthening (strain) which occurs during braking muscle actions at each landing phase in DR^3, 10, 13^, leading to disruptions of weaker sarcomeres and/or excitation-contraction coupling failure^14, 15^. It should be noted that level^16^ or uphill^17^ running could also induce some damage, especially when it is performed for extremely long duration (e.g. 24 h^16^). This may be partly because some degree of eccentric component, especially at the ankle joint to perform stretch-shortening cycle^2^, is involved in these exercises as well. Nevertheless, several studies have reported that muscle damage is specifically induced by DR but not by level or uphill running^10, 16^, or very little^13^, if any. With such a background, DR has often been adopted as an exercise model to induce damage in leg muscles, with particular focus on the knee extensors (KEs)^10, 12, 18, 19^. This is reasonable considering the KEs’ largest size among others and fundamental roles in daily and sports activities including running^20–22^. On the other hand, it is also reported that a loss of strength occurs in the plantar flexors (PFs) after trail DR^3^ or mountain ultra-marathon^4, 5^ to a similar or even greater extent (depending on measurement timing) to that in the KEs. Considering this, it is worth focusing on not only the KEs but also PFs in order to better understand the possible muscle damage manifestations in the leg muscles induced by DR.

The KEs, i.e. the quadriceps femoris, is composed of four muscles; the rectus femoris (RF), vastus intermedius (VI), vastus lateralis (VL), and vastus medialis (VM). The main PFs, i.e. the triceps surae, is composed of three muscles; the lateral gastrocnemius (LG), medial gastrocnemius (MG), and soleus (SOL). Among them, the RF, LG, and MG are bi-articular muscles that cross two joints (RF: hip and knee joints, LG and MG: knee and ankle joints). Thus, their functions and activation patterns during locomotion are more complex than the mono-articular muscles that span only one joint^22, 23^. In addition, these bi-articular muscles have a higher proportion of fast-twitch fibers than their synergists^24, 25^, which have been reported to be preferentially involved during eccentric contractions^26^ and thus more prone to damage^9, 27^. Consequently, the incidence of muscle strain injuries in sports, which are often induced during eccentric muscle actions, is higher in the bi- than the mono-articular muscles^21, 28^. It is worth noting that such muscle strain injuries frequently occur near the muscle belly (~middle site along the muscles), rather than near the proximal or distal sites^21, 28^. In previous studies on DR-induced muscle damage, however, little attention has been paid on which site of the muscles, if any, is more damaged than others within a muscle group. For example, most studies on DR-induced muscle damage have used such indirect parameters as changes in strength, muscle soreness of a whole muscle or area (e.g. anterior thigh), and/or plasma CK activity as indices of muscle damage^10, 12, 18, 19^. While often used in related studies, none of these can explore which individual muscle was more affected by an exercise task. Some studies measured an electromyogram (EMG) during trail DR^3^ or took a muscle biopsy after treadmill DR^12^ from one muscle (i.e. the VL) as a representative of the KEs. However, it is unknown if muscle damage induced by DR occurs uniformly within the KEs or PFs.

While muscle damage can be directly observed by histological analyses of muscle biopsies^29^, its invasive nature prohibits repetitive and/or multiple measurements. This is why previous studies are limited to applying muscle biopsy data from one muscle to that of a whole muscle group^29^, which may be an incorrect assumption. On the other hand, magnetic resonance imaging (MRI) non-invasively allows for spatially resolved analysis of muscle tissue, and can be safely used in repetitive measurements. More specifically, transverse relaxation time (T_2_)-weighted MRI can provide information on water content of muscle tissue given as a T_2_ value, which can be then used as an index of the inflammatory edema induced by eccentric exercise^30, 31^. It has been reported that T_2_ increases immediately post-exercise regardless of muscle damage^31–35^ and subsides within as early as few minutes^36, 37^, and it increases again ≥~12 h post-exercise if muscle is damaged^36, 37^. For example, Ochi *et al*.^36^ reported that T_2_ significantly increased immediately post-exercise in the elbow flexors of both arms that performed either maximal concentric or eccentric contractions (5 sets × 6 reps), but T_2_ returned to baseline at 1 day post-exercise in the concentrically exercised arm (without DOMS) while it remained elevated at 1, 3, and 5 days post-exercise in the eccentrically exercised arm (with DOMS). Thus, it is considered that the acute T_2_ increase is associated with muscle activation during exercise^31–35^, and the delayed T_2_ increase is a consequence of muscle damage (inflammatory edema), the degree of which is often greater than the acute one^31, 32, 37^. To support the usefulness of T_2_ as an index of muscle damage, high correlation (*r* > 0.9) has been reported between changes in T_2_ and plasma CK activity measured within several days after eccentric exercises^30, 38^. Some studies demonstrated that T_2_ increased heterogeneously within the KEs^11, 31, 37^ or PFs^39, 40^ after eccentric or strenuous resistance exercises. To the authors knowledge, however, no studies have examined localization of muscle damage within the KEs and PFs induced by DR. Clarifying this would provide insight into which site or muscle should be given particular care and/or focus when conducting DR in a practical or experimental setting.

In this study, we aimed to identify which sites within the KEs and PFs are more damaged by DR by using T_2_-weighted MRI. For this purpose, we measured DR-induced changes in T_2_ values at proximal, middle, and distal sites of each of the KEs and PFs. Based on the aforementioned differences in functions and injury rates between the bi- and mono-articular muscles, we hypothesized that localization of damage (an increase in T_2_) would be more pronounced in the middle site of the RF among the KEs, and middle site of the LG or MG among the PFs. In addition, we measured maximal isometric torque, rate of torque development (RTD), and muscle soreness as indirect indices of damage^3–5, 9, 11, 12, 41, 42^. Given that the knee joint performs greater negative (eccentric) work than the ankle joint does during DR^2^, we also hypothesized that muscle damage would be greater for the KEs than the PFs.

## Methods

### Participants

A total of 16 young adults (8 males, 8 females) participated in this study (age: 24.9 ± 3.4 years, height: 1.68 ± 0.09 m, body mass: 60.8 ± 10.1 kg; means ± SDs). The participants were all healthy, but none had been involved in any type of systematic resistance, aerobic, or flexibility training program (≥30 min·day^−1^, ≥2 days·week^−1^), or had experienced strenuous mountain trekking and/or downhill walking/running (other than those encountered in daily activities) in the past 12 months. This study was approved by the Ethics Committee of the Waseda University and was consistent with institutional ethical requirements for human experimentation in accordance with the Declaration of Helsinki. Prior to any measurements, the participants visited the laboratory and were fully informed about the procedures and possible risks involved as well as the purpose of the study, and written informed consent was obtained. Before (pre) and 24, 48, and 72 h after DR, measurements of T_2_-MRI, muscle soreness, isometric torque, and RTD, in this order, were performed on the right limb. We did not perform measurements immediately post-exercise for the following reasons. 1) Delayed (≥24 h) T_2_ increase better reflects muscle damage than acute T_2_ increase does^37, 38^, as explained earlier. 2) In a typical safety guideline for MRI scanning^43^, it is advised to avoid sweating in a magnet bore since it increases the risk of burns by acting as the conductors. 3) Access to MR device (available time) was limited. Mainly for these reasons, it is common to conduct measurements at ≥24 h post-exercise in studies using T_2_-MRI^11, 30, 44, 45^ to quantify muscle damage. In addition, although some studies using single-joint maximal eccentric contractions, especially on arm muscles which are more vulnerable than leg muscles^46^, have reported symptoms of muscle damage lasting for >7 days^8, 45^, those using downhill running^10, 12^ or other exercises in leg muscles^4, 11, 12, 37, 39, 40, 42^ have often shown that muscle damage peaks and/or subsides within 72 h post-exercise. The DR and measurements were conducted as follows.

### DR

DR was performed on a treadmill (Mercury, h/p cosmos, Germany) with −15% slope for 45 min at each participant’s maximal tolerable velocity. The laboratory temperature was 20–25 °C and relative humidity was 40–60%. As a warm-up and familiarization, the participants first walked at 1 km/h on the −15% slope and gradually increased the velocity until the maximal tolerable velocity (the velocity beyond which the participants felt unsustainable for 45 min) was determined. After a warm-up of ~5 min of sub-maximal running at a jogging velocity followed by ~3-min rest, the participants performed the 45-min DR task under the supervision of at least one of the authors. During the task, the participants were periodically asked (every ~5 min) if the velocity was their maximal tolerable or not, and it was corrected when necessary. Participants wore their own sports shoes and clothes but were not allowed to wear any compression garments during the DR. Participants were instructed not to perform any unfamiliar activities or any interventions that could affect the recovery such as, but not limited to, massage, icing, and nutritional supplementations during the experimental period.

### Maximal isometric torque and RTD

By using specially designed rigid isometric torque dynamometers (VTK-002 and VTF-002, Vine, Japan), maximal isometric torque and RTD of the KEs and PFs were measured. For the KEs measurement, the participants sat on the device with hip and knee joint angles fixed at 80° and 70° (anatomical position = 0°), respectively, and an adjustable strap was tightly fastened across the pelvis to prevent extraneous movement. The peak isometric knee extension torque has been reported to occur at ~70° knee joint angle^47^, and our previous study^11^ using the same device and posture as used in this study found a significant decrease in maximal torque at 24 h after eccentric exercises of the KEs. For the PFs measurement, the participants sat on the device with hip, knee, and ankle angles fixed at 80°, 0°, and 0°, respectively, and adjustable straps were tightly fastened across the thigh and foot. Although peak isometric plantar flexion torque occurs at a more dorsiflexed position (~−15°)^48^, a previous study^39^ using the same device and posture as used in this study found a significant decrease in maximal torque at 24 and 48 h after strenuous (damaging) exercise of the PFs. The measurements were done in the order of the KEs and PFs. The torque signals were amplified by a strain amplifier (DPM-711B, Kyowa, Japan) and analogue-digital converted (Power Lab, ADInstruments, Australia) into a computer at 1,000 Hz. Prior to each measurement, participants performed an adequate warm-up, consisting of submaximal contractions of 30%, 50%, and 80% of maximal effort. After at least a 1-min rest, participants performed two ramp maximal voluntary contractions with a 1-min rest in-between trials. Participants were asked to develop torque gradually over 5 s to reach maximum and then to sustain maximal effort for ~3 s, with verbal encouragement provided by the examiner. Additional trials were performed if the difference in the peak torque of the two trials was more than 10%. In this study, the participants performed 2–4 trials in total at each session (for each of the KEs and PFs). Previous studies on muscle damage in the KEs and/or PFs often report >10% decrease at 24, 48, and/or 72 h post-exercise^3, 10, 39, 42^, and 10% cut-off used in this study has been shown to detect a significant decrease in maximal torque of the KEs or PFs at 24, 48, and/or 72 h after eccentric or strenuous exercise in previous studies^11, 39, 42^. The greatest instantaneous torque achieved during the two MVC trials or subsequent explosive contraction trials (explained below) was adopted as the maximal torque.

After the MVC trials, participants performed 10 explosive voluntary contractions, preceded by familiarization with the task (2–4 contractions). The task and analysis were performed in accordance with recommendations by a recent review^49^. The participants were instructed to perform each contraction “as fast and hard as possible” for 1 s, giving particular emphasis on “fast” contraction, with a 20-s rest between contractions. Contractions with a visually detectable change in baseline torque (pre-tension or countermovement) prior to contraction onset were discarded. The three contractions with the highest peak slope, corresponding to peak RTD during a contraction, were analyzed in detail using Matlab software. RTD was measured as the slope of the torque-time curve in the time intervals of 0–30 ms (RTD_30_), 0–50 ms (RTD_50_), 0–100 ms (RTD_100_), and 0–200 ms (RTD_200_) from contraction onset, and the average of the three trials was taken. Contraction onset was defined as the time point at which the torque curve exceeded baseline torque by more than 1% of maximal torque for each day of the measurements.

### T_2_-MRI

**Figure 1:**
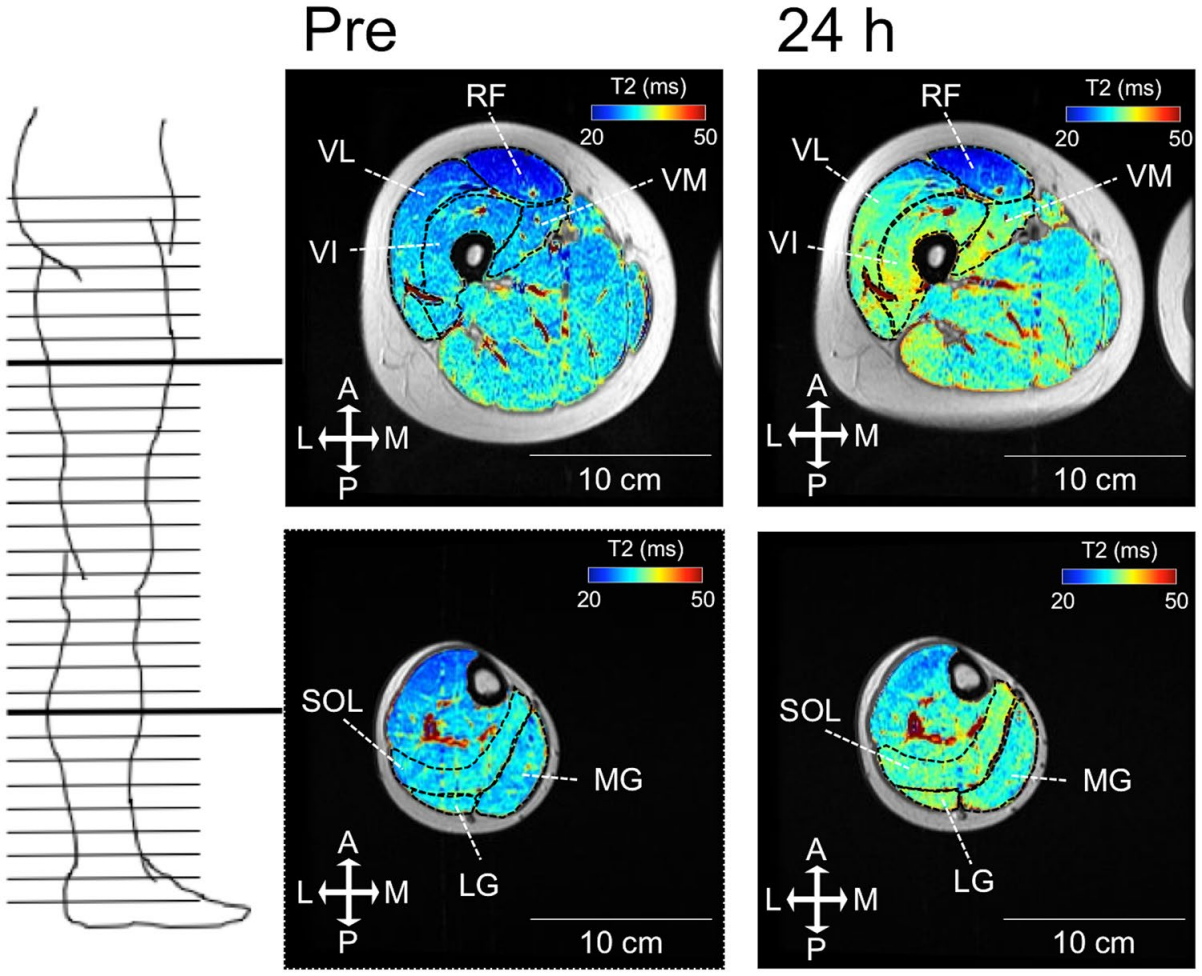
Examples of T_2_ maps superimposed on T_2_-MRIs at middle sites for the VI (KEs, upper figures) and SOL (PFs, lower figures) scanned before (pre, left) and 24 h after (right) the downhill running task for one participant are shown. T_2_: transverse-relaxation time, MRI: magnetic resonance imaging, RF: rectus femoris, VI: vastus intermedius, VL: vastus lateralis, VM: vastus medialis, LG lateral gastrocnemius, MG medial gastrocnemius, SOL: soleus, A, M, P, L: anterior, medial, posterior, and lateral side, respectively.

T_2_-weighted MRIs (echo times: 25, 50, 75 and 100 ms, repetition time: 2000 ms, matrix: 256 × 256, field of view: 24 cm, slice thickness: 1.5 cm, gap: 1 cm) of the whole right leg, with two scans for the upper (KEs) and lower (PFs) leg, in the transverse plane were recorded. Participants lay supine with their legs fully extended and muscles relaxed in a magnet bore (Signa EXCITE 1.5 T, GE Medical Systems, USA). A specific slice was always set using the same anatomical markers (i.e. the first slice at the proximal edge of the femoral head for the upper leg scan, and the third slice at the tibia condyle for the lower leg scan). Images were analyzed with ImageJ software (National Institute of Health, USA). Regions of interest were drawn in each slice by manually tracing the border of the anatomical cross-sectional area at proximal (25%), middle (50%), and distal (75%) sites of muscle length for each of the KEs (RF, VI, VL, VM) and PFs (LG, MG, SOL) (Fig. 1). Care was taken to exclude visible adipose and connective tissue incursions. T_2_ relaxation time was calculated by least-squares analysis, fitting the signal intensity at each echo time (25, 50, 75 and 100 ms) to a monoexponential decay.

### Muscle soreness

Muscle soreness in the anterior thigh and the posterior lower leg was assessed using a 0–10 scale (0 = no pain, 10 = maximal pain) as the pain perceived during a squat and a calf raise movement. Participants were instructed to perform a squat (knee flexed until ~90°) with the feet shoulder-width apart and subsequently calf raise (to near-maximal height) movements 2–3 times without a counter-movement, from which they rated the level of soreness in 0–10 scale for each muscle group. Based on the Universal Pain Assessment Tool (http://pronursingservice.com), which is often used in a clinical setting, the severity of muscle soreness was categorized as no (<1), mild (1–2.9), moderate (3–6.9), and severe (≥7).

### Reproducibility of the measurements

Day to day (separated by 1–4 days) reproducibility of the measurements was examined on 10 participants for all variables except for soreness. Paired t-tests revealed no significant differences between days in all the variables. The coefficient of variation (CV) and the intraclass correlation coefficient (ICC) for each measurement variable are shown in Table 1. Both the CV and ICC have been widely used to assess reproducibility, and provide a measure of absolute (within-individual) and relative (inter-individual) reliability, respectively^50^. The CV was low for maximal torque (≤5.3%) and T_2_ (≤2.0%) but relatively high for the RTD (≥6.3%) especially in the early phase (RTD_30, 50_: 17.2–21.1%). ICC values were interpreted as excellent: 0.80–1.00, good: 0.60–0.80, and poor: <0.60^51^, and all variables ranged from good to excellent (0.70–0.95).

**Table 1:** The coefficient of variations (CVs) and intraclass correlation coefficients (ICCs) of the variables in between-days measurements (n = 10).

Dependent variables	CV (%)		ICC	
	KE	PF	KE	PF
Maximal torque	4.6	5.3	0.92	0.95
RTD_30_	18.4	21.1	0.74	0.68
RTD_50_	17.2	18.7	0.81	0.77
RTD_100_	7.4	10.5	0.90	0.83
RTD_200_	6.3	8.8	0.88	0.82
T_2_	≤1.7	≤2.0	≥0.75	≥0.70

KE: knee extensors, PF: plantar flexors, RTD_30, 50, 100, 200_: rate of torque development in the time intervals of 0–30, 0–50, 0–100, and 0–200 ms, respectively, from contraction onset. T_2_: transverse-relaxation time. CVs and ICCs for the T_2_ are maximum and minimal values, respectively, among the 21 sites.

### Statistical analysis

Descriptive data are presented as means ± SDs. All data were analyzed using SPSS software (version 24.0, IBM Corp, USA). Changes in each of maximal torque, RTD, and soreness were compared by a two-way (4 time points × 2 muscle groups) repeated measures analysis of variance (ANOVA). When a significant interaction effect was found, a one-way ANOVA (4 time points) followed by a Dunnett’s post hoc test were performed to compare changes from pre for each muscle group. In addition, within-individual changes from pre were calculated for each time point after exercise, and they were compared among muscle groups by a two-way ANOVA (3 time points × 2 muscle groups). To examine if pre values influenced magnitudes of changes, Pearson’s correlations between pre values and changes were examined for maximal torque and RTD. Changes in T_2_ were compared by a two-way (4 time points × 21 sites) ANOVA. When a significant interaction was found, a one-way ANOVA (4 time points) followed by a Dunnett’s post hoc test were performed to compare changes from pre for each site. In the same way as above, within-individual changes from pre were calculated for each time point after exercise, and they were compared among sites by a two-way ANOVA (3 time points × 21 sites). As indices of effect size (for post hoc comparisons), Cohen’s *d* was calculated and interpreted as large: ≥0.80, medium: 0.50–0.79, small: 0.20–0.49, and trivial: <0.20^52^. To describe relationships between changes in functional performances (maximal torque and RTD) and T_2_ in each of the KEs and PFs, Pearson’s correlations were calculated. Sphericity was checked by Mauchly’s test in ANOVA and *P* values were modified with Greenhouse-Geisser correction when necessary. Statistical significance was set at *P* < 0.05.

## Results

### DR

The mean velocity of running was 10.0 ± 1.4 km/h. Of 16 participants, 15 completed the 45-min DR task. One participant discontinued running at 25 min due to pain in a toenail developed during running. Another participant, who completed the 45-min DR, could not perform strength measurements post-exercise due to right ankle joint pain developed after running. We excluded these 2 participants from the analysis to avoid any confounding factors. Consequently, the number of the participants in the following results section is 14 for all variables.

### Maximal torque and RTD

**Figure 2:**
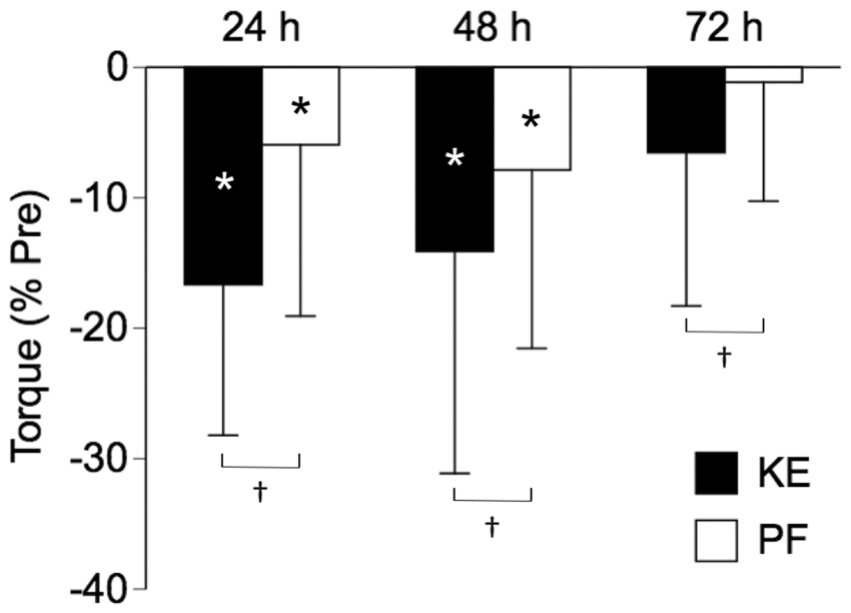
Relative changes from pre in maximal isometric torque at 24, 48, and 72 h after downhill running. KE: knee extensors, PF: plantar flexors. Values are means ± SDs. *Significantly different from pre at *P* < 0.05 based on absolute changes shown in Table 2. ^†^Significantly different between muscle groups based on relative changes at *P* < 0.05.

Table 2 shows absolute changes in maximal torque and RTD. A significant main effect of time point (*P* < 0.001), without a significant main effect of muscle group (*P* = 0.106) or their interaction (*P* = 0.077), was found in the maximal torque. Combining data from both muscle groups, a one-way ANOVA (*P* < 0.001) followed by a Dunnett’s post hoc test found significant decreases in the maximal torque at 24 (*P* < 0.001, *d* = 0.45) and 48 (*P* < 0.001, *d* = 0.38) h for this pooled data. A significant time point-muscle group interaction (*P* = 0.013) was found in the RTD_30_. A one-way ANOVA (*P* = 0.04 for KEs and 0.01 for PFs) followed by a Dunnett’s post hoc test for each muscle group found significant decreases in the RTD_30_ for KEs at 24 (*P* = 0.001, *d* = 0.59), 48 (*P* = 0.049, *d* = 0.32), and 72 (*P* = 0.022, *d* = 0.37) h, and for PFs at 48 (*P* = 0.017, *d* = 0.45) and 72 (*P* = 0.009, *d* = 0.46) h. RTD_50_ and RTD_100_ found a significant main effect of time point (*P* = 0.016 for RTD_50_ and 0.007 for RTD_100_) and muscle group (*P* = 0.003 and < 0.001), without a significant interaction (*P* = 0.053 and 0.064). Combining data from both muscle groups, a one-way ANOVA (*P* < 0.001 and < 0.001) followed by a Dunnett’s post hoc test found significant decreases in RTD_50_ at 24 (*P* < 0.001, *d* = 0.37), 48 (*P* = 0.007, *d* = 0.27), and 72 (*P* = 0.002, *d* = 0.30) h, and in RTD_100_ at 24 (*P* < 0.001, *d* = 0.38), 48 (*P* = 0.001, *d* = 0.31), and 72 (*P* = 0.005, *d* = 0.32) h for this pooled data. A significant time point-muscle group interaction (*P* = 0.040) was found in the RTD_200_. A one-way ANOVA found a significant main effect of time in KEs (*P* < 0.001) but not in PFs (*P* = 0.076). Dunnett’s post hoc test for KEs found significant decreases in the RTD_200_ at 24 (*P* < 0.001, *d* = 0.50), 48 (*P* < 0.001, *d* = 0.40), and 72 (*P* = 0.031, *d* = 0.27) h. For the relative changes (% Pre), a significant main effects of time point (*P* = 0.002) and muscle group (*P* = 0.042), without their interaction (*P* = 0.341), were found for maximal torque, indicating that relative reductions in maximal toque were significantly greater for the KEs than PFs at all time points post-exercise (Fig. 2). Relative changes of RTD (% Pre) in all phases did not show a significant main effect or interaction (*P* ≥ 0.099). No significant correlations between pre values and changes were found in any of the maximal torque and RTD for both KEs and PFs (*P* ≥ 0.121).

**Table 2:** Maximal torque (Nm), RTD (Nm/s), and soreness before and 24, 48, and 72 h after downhill running.

		Pre	24 h	48 h	72 h
Torque_max_	KE	210.5 ± 61.1	176.7 ± 62.7***	183.0 ± 69.0***	198.3 ± 67.2
	PF	179.8 ± 48.4	166.0 ± 33.1***	164.7 ± 42.0***	177.4 ± 46.4
RTD_30_	KE	553.5 ± 471.1	322.2 ± 300.4**	406.1 ± 439.5*	386.4 ± 431.9*
	PF	199.4 ± 101.5	169.9 ± 85.0	157.7 ± 85.6*	154.4 ± 92.6**
RTD_50_	KE	762.2 ± 591.9	497.3 ± 405.0***	582.0 ± 491.9**	556.8 ± 477.5**
	PF	280.2 ± 153.1	237.5 ± 118.7***	221.8 ± 124.7**	217.8 ± 136.4**
RTD_100_	KE	856.4 ± 471.3	640.2 ± 339.5***	701.3 ± 402.3***	694.7 ± 364.4
	PF	437.6 ± 210.6	380.9 ± 160.4***	354.8 ± 186.0***	360.1 ± 215.5***
RTD_200_	KE	710.3 ± 304.8	574.3 ± 232.9***	595.1 ± 264.3***	633.1 ± 261.3*
	PF	475.0 ± 148.2	430.2 ± 126.2	408.1 ± 140.0	426.1 ± 182.2
Soreness	KE	0.4 ± 0.9	6.5 ± 2.4***	6.6 ± 2.4***	3.9 ± 2.0***
	PF	0.4 ± 0.7	6.3 ± 2.5***	6.9 ± 1.6***	4.1 ± 1.8***

*, **, ***:Significantly different from pre at *P* < 0.05, *P* < 0.01, and *P* < 0.001, respectively. Torque_max_: maximal torque, KE: knee extensors, PF: plantar flexors, RTD_30, 50, 100, 200_: rate of torque development in the time intervals of 0–30, 0–50, 0–100, and 0–200 ms, respectively, from contraction onset.

### Muscle soreness

A significant main effect of time point (*P* < 0.001), without a significant main effect of muscle group (*P* = 0.895) and their interaction (*P* = 0.702), was found in muscle soreness. A one-way ANOVA (*P* < 0.001) followed by a Dunnett’s post hoc test on pooled data found significant increases from baseline at 24 (*P* < 0.001, *d* = 3.35), 48 (*P* < 0.001, *d* = 4.17), and 72 (*P* < 0.001, *d* = 2.53) h post-exercise (Table 2). Relative changes (Δ Pre) also did not find a significant main effect of muscle group (*P* = 0.793) or interaction (*P* = 0.428).

### T_2_-MRI

**Figure 3:**
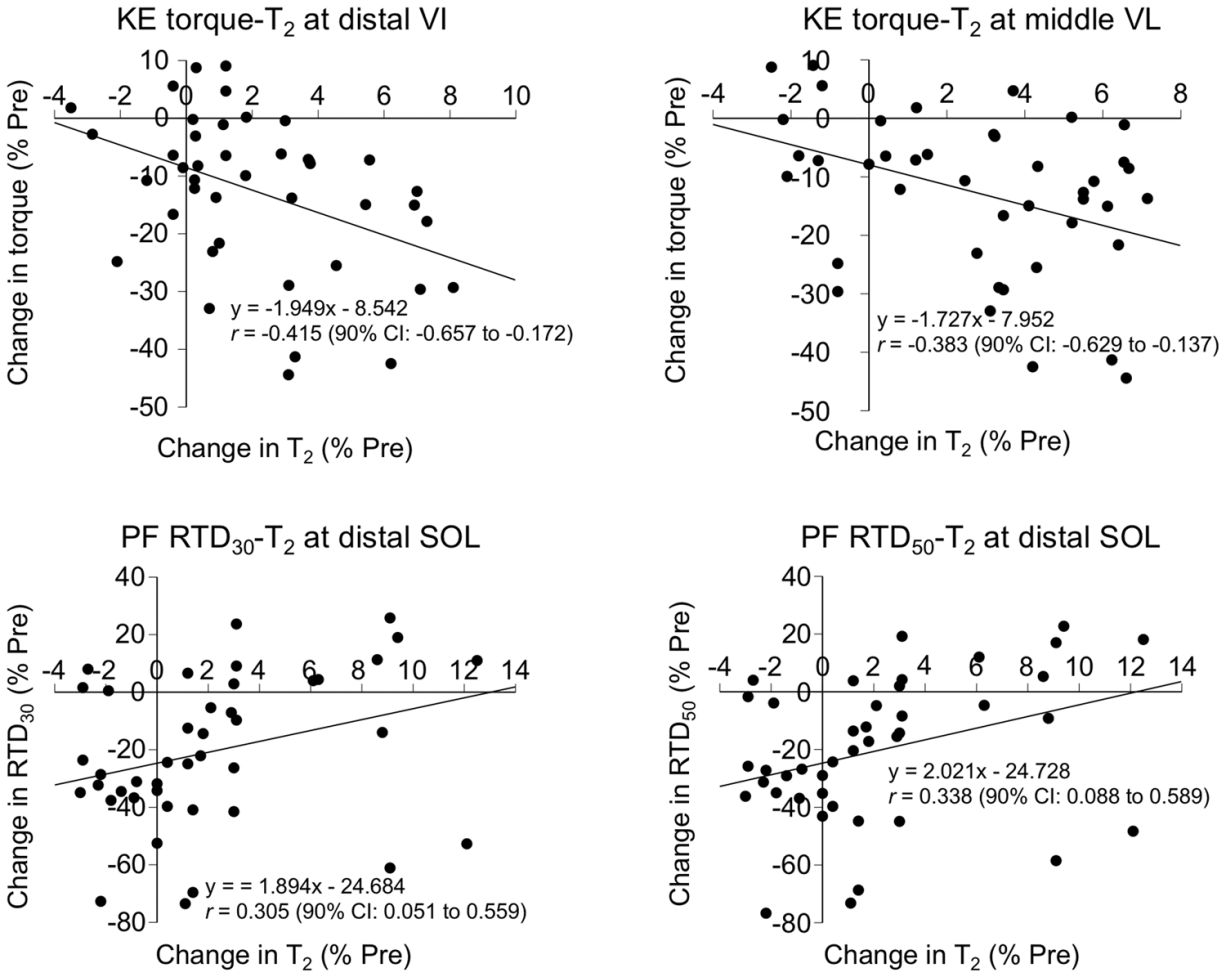
Relationships between changes in maximal torque of the KEs and T_2_ at the distal VI (top left) and at the middle VL (top right), between changes in RTD_30_ of the PFs and T_2_ at the distal SOL (bottom left), and between changes in RTD_50_ of the PFs and T_2_ at the distal SOL (bottom right). KEs: knee extensors, PFs: plantar flexors, RTD_30,50_: rate of torque development in the time intervals of 0–30 and 0–50 ms, respectively, from contraction onset. Each graph has 42 plots (14 participants × 3 time points post-exercise).

A significant time point-site interaction (*P* = 0.010) was found in the T_2_ changes (Table 3). A one-way ANOVA followed by a Dunnett’s post hoc test for each site showed that T_2_ significantly increased at the proximal VI at 24 h (*P* = 0.004, *d* = 1.05), middle VI at 24 (*P* < 0.001, *d* = 1.64) and 48 (*P* < 0.001, *d* = 1.13) h, middle LG at 24 h (*P* = 0.020, *d* = 0.76), proximal SOL at 24 h (*P* = 0.004, *d* = 0.77), and middle SOL at 48 h (*P* = 0.023, *d* = 0.72). Among the sites, the peak Cohen’s *d* was large (≥0.8) at the proximal (1.05) and middle (1.64) VI. Relative changes (ΔPre) found a significant time point-site interaction (*P* < 0.001), but subsequent one-way ANOVA did not find a significant difference between sites at each time point post-exercise (*P* ≥ 0.209). Significant negative correlations were found between changes in maximal torque of the KEs and T_2_ at the distal VI (Fig. 3, top left, *P* = 0.006) and middle VL (Fig. 3, top right, *P* = 0.012). In contrast, significant positive correlations were found between changes in RTD_30_ of the PFs and T_2_ at the distal SOL (Fig. 3, bottom left, *P* = 0.495), and between changes in RTD_50_ of the PFs and T_2_ at the distal SOL (Fig. 3, bottom right, *P* = 0.028).

**Table 3:** T_2_ values (ms) measured before and 24, 48, and 72 h after downhill running.

		Pre	24 h	48 h	72 h	Peak *d*
RF	Proximal	24.8 ± 1.5	25.0 ± 1.4	24.9 ± 1.6	24.9 ± 1.4	0.15
	Middle	24.8 ± 1.6	25.1 ± 1.9	25.1 ± 1.6	25.1 ± 1.3	0.24
	Distal	25.1 ± 1.4	25.6 ± 1.3	25.1 ± 1.4	25.7 ± 1.5	0.40
VI	Proximal	28.9 ± 1.3	30.1 ± 1.2**	29.3 ± 1.5	29.5 ± 0.9	1.05^†^
	Middle	29.7 ± 0.9	31.1 ± 0.7***	30.8 ± 1.0***	30.3 ± 0.8	1.64^†^
	Distal	30.1 ± 1.0	30.7 ± 1.0	30.4 ± 1.3	30.5 ± 0.9	0.69
VL	Proximal	28.6 ± 1.5	29.5 ± 1.2	29.4 ± 1.2	29.1 ± 0.9	0.68
	Middle	29.7 ± 1.5	30.5 ± 1.3	30.5 ± 1.0	30.1 ± 1.2	0.62
	Distal	29.7 ± 1.4	30.1 ± 1.1	30.2 ± 1.2	29.7 ± 0.9	0.34
VM	Proximal	29.9 ± 1.2	30.4 ± 1.5	30.5 ± 1.6	30.1 ± 1.1	0.41
	Middle	30.4 ± 1.6	31.1 ± 1.1	31.1 ± 1.3	30.6 ± 1.0	0.53
	Distal	29.4 ± 1.1	30.3 ± 1.3	30.0 ± 1.0	29.9 ± 0.9	0.71
LG	Proximal	30.4 ± 2.2	31.8 ± 2.7	31.4 ± 2.5	30.1 ± 1.9	0.55
	Middle	30.6 ± 1.9	32.2 ± 2.2*	31.4 ± 1.9	30.6 ± 1.3	0.76
	Distal	33.8 ± 3.7	34.6 ± 2.9	35.0 ± 3.7	32.9 ± 1.9	0.52
MG	Proximal	29.3 ± 1.9	30.1 ± 1.7	30.4 ± 2.3	29.1 ± 2.2	0.72
	Middle	30.9 ± 1.9	31.4 ± 1.6	31.4 ± 1.8	30.7 ± 1.3	0.49
	Distal	33.0 ± 2.8	33.7 ± 2.5	34.3 ± 3.2	32.4 ± 1.8	0.70
SOL	Proximal	31.3 ± 1.5	32.6 ± 1.8**	31.9 ± 1.7	31.6 ± 1.6	0.77
	Middle	33.2 ± 1.5	34.2 ± 1.9	34.4 ± 1.6*	33.2 ± 2.1	0.72
	Distal	34.1 ± 1.6	34.9 ± 2.0	34.3 ± 1.6	34.0 ± 1.0	0.28

*, **, ***:Significantly different from pre at *P* < 0.05, *P* < 0.01, and *P* < 0.001, respectively. ^†^The sites where Cohen’s *d* regarding T_2_ change from pre was large (*d* ≥ 0.8). RF: rectus femoris, VI: vastus intermedius, VL: vastus lateralis, VM: vastus medialis, LG: lateral gastrocnemius, MG: medial gastrocnemius, SOL: soleus.

## Discussion

The main findings of the present study were that 1) DR induced significant decreases in maximal torque in both KEs and PFs at 24 and 48 h post-exercise, with greater reductions for KEs than PFs, 2) RTD in all phases significantly decreased at all time points post-exercise in KEs, while RTD_50_ at 24 h and RTD_200_ at all time points did not significantly decrease in PFs, and 3) T_2_ significantly increased at several sites of the KEs and PFs at 24 and/or 48 h post-exercise, with pronounced effect size found at the proximal and middle VI compared to the other sites. These results refuted our first hypothesis that a T_2_ increase would be pronounced at the middle site of the bi-articular muscles (RF and LG/MG), while at least indirectly supporting the second hypothesis that DR would induce greater damage in the KEs than PFs. Collectively, these results suggest that DR induces damage in both KEs and PFs, and especially affects the proximal and middle sites of the VI in the KEs, while damage is relatively sporadic in the PFs.

DR induced significant decreases in maximal torque of both of the KEs and PFs (Table 2). The reductions in maximal torque of the KEs in this study were −17% at 24 h and −14% at 48 h post-exercise (Fig. 2), which were similar to the report of Malm *et al*.^12^ (−17% at 24 h and −12% at 48 h post-exercise) or Eston *et al*.^53^ (~−20% at 24 h post-exercise; visually estimated from the Fig. 2 of their study) following treadmill DR. Giandolini *et al*.^3^ reported reductions of maximal torque of −10% in the PFs at 48 h after trail DR, which was also comparable to our result (−8% at 48 h post-exercise). Thus, it is safe to assume that typical muscle damage was induced in both KEs and PFs by DR performed in this study. The result on muscle soreness (i.e. peak: ~7 out of 10; ~severe pain) (Table 2), while subjective, also indicates that muscle damage occurred in both muscle groups. In addition, this study revealed that DR induced significant decreases in RTD in all phases at all time points post-exercise in both muscle groups, except for RTD_50_ at 24 h and RTD_200_ at all time points post-exercise in PFs (Table 2). These results, together with the greater reductions of maximal torque for the KEs than PFs (Fig. 2), collectively suggest that DR induced damage in both muscle groups, with greater degree for the KEs than PFs. This is reasonable since the knee joint performs the highest negative (eccentric) work (~63% of the total negative work) during DR, followed by the ankle (~23%) and hip (~15%) joints^2^. The reductions in muscle functions following DR has been attributed to both central and peripheral factors^1, 3, 54^. For example, it is reported that DR induced a significant reduction in maximal torque of the KEs^54^ or both of the KEs and PFs^3^, accompanied with reduced neural drive (as assessed by voluntary activation level) and impaired contractile properties (as assessed by evoked torque, M-wave amplitude, and/or low-frequency fatigue), with greater changes in the latter (peripheral factors). Although we do not have any data on either central or peripheral factors explaining our results, the observed changes in maximal torque and RTD in this study could be attributed to both central and peripheral factors, with a particular emphasis on the latter. This issue requires further investigation utilizing such techniques as transcranial magnetic stimulation and peripheral nerve stimulation^6, 7^.

Significant negative correlations were found between changes in maximal torque of the KEs and T_2_ at the distal VI and at the middle VL (Fig. 3, top left and right). This indicates that the more T_2_ increased, the more strength decreased, supporting the usefulness of T_2_ as an index of muscle damage. To our knowledge, this is the first study to show such relationships between changes in functional performance and T_2_. However, the significant but weak to moderate correlations (*r* = −0.383–0.415) indicate that only a very small portion (15–17%; *r* ^*2*^ = 0.147–0.173) of the strength reduction can be explained by a T_2_ change in one site of the muscles. This would be mainly due to the fact that the torque output is the sum of the torque produced by each synergist. On the other hand, significant positive correlations were found between changes in the T_2_ at the distal SOL and RTD_30_ as well as RTD_50_ of the PFs (Fig. 3, bottom left and right). This result is surprising, and a physiological background for this is unclear. Although purely speculative, inter-muscular compensations may partly account for the result. It is reported that when one muscle (e.g. SOL) is fatigued, the activity of synergistic muscle (e.g. LG or MG) increases^55^. Since the SOL is rich in slow-fiber^24, 25^, it is possible that the more the SOL was damaged, the more the other PFs (LG or MG), which are more rich in fast-fiber than SOL^24, 25^, were recruited, contributing to retaining RTD. However, it should be noted that the reproducibility of the early-phase RTD (RTD_30_ and RTD_50_) was low (inter-day CV = 17.2–21.1%) compared to those of the maximal torque (≤5.3%) and T_2_ (≤2%) for both muscle groups, which is in line with previous reports^44, 49^. Thus, the coefficient of determinations (9–10%; *r* ^*2*^ = 0.091–0.102) between changes in T_2_ and RTD may be negligible.

T_2_ significantly increased at the proximal and middle VI, middle LG, and proximal and middle SOL. Among the sites, effect size analysis showed that T_2_ changes were prominent at the proximal (1.05) and middle (1.64) VI compared to those of the PFs (0.72–0.77). This result did not support our hypothesis that muscle damage would be greater in the middle site of the bi-articular muscles (i.e. RF, LG/MG). We set this hypothesis partly based on the fact that eccentric muscle action-induced strain injuries in sports often occur near the muscle belly (i.e. ~middle site) of the bi-articular muscles^21, 56^. However, it should be borne in mind that there are various types of eccentric movement patterns that would cause strain injuries in sports, such as a rapid deceleration or change of direction in a difficult body position, sprinting, and/or kicking a ball^21, 56^. These are more complex in executing the movement and probably more intense (stressful) per one action compared to DR, which was tolerable for a certain period of time (e.g. 45 min in this study). Such differences in movement complexity and/or instantaneous stress would at least in part account for the difference in the most affected sites between the strain injuries in sports and DR-induced muscle damage.

Then, why was the damage pronounced in the VI within the KEs? One possible explanation may be related to a load sharing during exercise^57^. It is known that each muscle among synergists differently contributes to torque output, and the contribution of the VI has been reported to be much higher (≥40% of total output) than the other KEs (~10–25% for each muscle) during submaximal isometric contractions (≤50% maximal voluntary contraction)^57^. Although this finding is not directly applicable to our results given the difference in the muscle contraction types between studies, it is possible that the VI contributed more than the other KEs during DR, since the KEs performed submaximal contractions throughout the 45-min DR trial. Regarding the PFs, a computational modeling study^58^ suggested a greater contribution of SOL than the others in the early stance (braking) phase of level running. However, it is unknown whether the same is true for DR, and indeed our results suggest a similar degree of muscle damage among the middle LG, proximal SOL, and middle SOL (*d*: 0.72–0.77). More research is needed to identify an association between contribution/activation of each muscle during DR and resultant muscle damage.

Significant and pronounced T_2_ increase was found at the proximal and middle VI, but not at the distal VI. Furthermore, it is worth noting that while T_2_ significantly increased at the proximal and/or middle sites of other muscles, no such change was observed at the distal site of any muscles (Table 3). This suggests that the distal site is less susceptible to damage, regardless of the muscles. The reason for this is unclear, but may be related to a difference in mechanical/material properties along the muscles. The KEs and PFs merge with the patellar (quadriceps) and Achilles tendons, respectively, at their distal sites. Both of these tendons are known to have much higher stiffness (or Young’s modulus) than the muscles, enabling transmission, attenuation, and conservation of high muscle forces, as well as protection against muscle damage by its buffer-like behavior^59^. Considering this, together with the fact that muscle damage is a function of active muscle strain^14^, it is possible that the degree of active strain would be less at the distal sites of the muscles, owing to tendon properties, compared to the proximal and middle sites, which are more likely to be affected by the muscle belly^20, 60^. Further research exploring fascicle behavior during DR is necessary to clarify such intra-muscle differences in the muscle damage responses.

We did not set a level running condition as a control group, so how much of the observed results is attributable to “downhill” effect is unknown. However, as mentioned earlier, it is reported that while DR induced significant changes in muscle damage markers, level running matched for relative intensity and duration did not^10^, and that even maximal concentric contractions of the elbow flexors did not induce T_2_ change at ≥24 h post-exercise (i.e. little damage)^36^. It is also worth noting that although muscle damage was evident in this study based on the decreases in maximal torque and RTD as well as occurrence of DOMS, the observed increases in T_2_ were relatively small (3–5%). Importantly, these values were within the range of those (3–11%) reported by previous studies^11, 37, 61^ on a T_2_ change in the KEs following multi-joint eccentric exercises. This suggests that a T_2_ response is generally low compared to other muscle damage markers, at least in the leg muscles. Based on these, it seems unlikely that level running induces significant T_2_ increases in the KEs or PFs, unless it is performed for extremely long duration or distance^16^. In this sense, it is yet to be explored whether localization of muscle damage differs between DR and level or uphill running conditions, if the latter two were performed until the point where it causes significant T_2_ increases in the leg muscles. However, caution is warranted because when performing such extreme protocols with either level or uphill running, and especially with DR matched for duration and/or relative intensity, the risk of injury would be very high. Indeed, two participants in this study could not complete either the 45-min DR or post-exercise measurements due to the unbearable pain that occurred during or after the DR.

No data collections were made on biomechanical (e.g. kinematics) or physiological (e.g. ${\dot{{\rm{V}}}{\rm{O}}}_{{\rm{2}}}$) parameters during DR in this study, so their inter-individual differences or changes during the DR task are unknown. It is suggested that running technique such as a foot strike pattern or stride frequency is a factor associated with muscle damage^1^, which may consequently affect ${\dot{{\rm{V}}}{\rm{O}}}_{{\rm{2}}}$ during DR^19^. Clarifying an association between running kinematics and DR-induced muscle damage may facilitate more effective injury prevention and/or training guidance and prescription. It must be reminded that the DR in this study was conducted on a treadmill, so the results may not necessarily match those induced by DR in a real condition. Indeed, it is expected that running kinematics, especially a foot strike pattern, would vary at each step in real settings, increasing its variability and thus the stress particularly at the ankle joint level. Future studies should aim to address these issues and connect gaps between laboratory and real settings, for the better understanding of localization of muscle damage induced by DR.

In conclusion, 45-min downhill running performed at a maximal tolerable velocity induced significant reductions in muscle functions in both of the knee extensors and plantar flexors, with its influence greater for the former than the latter. Inflammatory edema, assessed by a T_2_ change, was pronounced at the proximal and middle sites of the vastus intermedius. These results suggest that the vastus intermedius, although often “not highlighted” possibly due to being a deeply located muscle, should also be included in examination whenever possible in future studies evaluating muscle damage induced by DR.
